# Imaging of Motor Cortex Physiology in Parkinson's Disease

**DOI:** 10.1002/mds.102

**Published:** 2018-10-02

**Authors:** Roxana G. Burciu, David E. Vaillancourt

**Affiliations:** ^1^ Department of Kinesiology and Applied Physiology University of Delaware Newark Delaware USA; ^2^ Department of Applied Physiology and Kinesiology University of Florida Gainesville Florida USA; ^3^ Department of Neurology University of Florida Gainesville Florida USA; ^4^ Department of Biomedical Engineering University of Florida Gainesville Florida USA

**Keywords:** Parkinson's disease, primary motor cortex, functional imaging, structural imaging, electrophysiology

## Abstract

There is abundant evidence that the pathophysiology of Parkinson's disease (PD) is not confined to the nigrostriatal dopaminergic pathway but propagates along the cortico‐basal ganglia‐thalamo‐cortical neural network. A critical node in this functional circuit impacted by PD is the primary motor cortex (M1), which plays a key role in generating neural impulses that regulate movements. The past several decades have lay witness to numerous in vivo neuroimaging techniques that provide a window into the function and structure of M1. A consistent observation from numerous studies is that during voluntary movement, but also at rest, the functional activity of M1 is altered in PD relative to healthy individuals, and it relates to many of the motor signs. Although this abnormal functional activity can be partially restored with acute dopaminergic medication, it continues to deteriorate with disease progression and may predate structural degeneration of M1. The current review discusses the evidence that M1 is fundamental to the pathophysiology of PD, as measured by neuroimaging techniques such as positron emission tomography, single‐photon emission computed tomography, electroencephalography, magnetoencephalography, and functional and structural MRI. Although novel treatments that target the cortex will not cure PD, they could significantly slow down and alter the progressive course of the disease and thus improve clinical care for this degenerative disease. © 2018 The Authors. Movement Disorders published by Wiley Periodicals, Inc. on behalf of International Parkinson and Movement Disorder Society

Cell death in the substantia nigra and the shortage of dopamine in the striatum have been known as key developments that affect motor control in Parkinson's disease (PD).^1^, ^2^, ^3^, ^4^, ^5^, ^6^, ^7^ Over time, however, it has become apparent that PD is associated with more complex changes across several key regions of the motor network, including the primary motor cortex (M1).^7^, ^8^, ^9^, ^10^ The dopaminergic cell loss in PD is believed to lead to an increased inhibition of the motor thalamic nuclei and decreased excitation of the cerebral cortex, which contributes to abnormal motor output.^11^, ^12^ There is also increasing evidence on abnormal neuronal oscillations within and between motor regions in PD, and these abnormal oscillations are thought to impair motor function and account for some of the clinical signs.^13^, ^14^, ^15^, ^16^

One way to probe in vivo the functional and structural integrity of key nodes of the cortico‐basal ganglia motor circuit is by using brain imaging and electrophysiological techniques.^8^, ^17^, ^18^ Therefore, the aim of this article is to provide a comprehensive overview of the involvement of M1 in the pathophysiology of PD as revealed by modern brain imaging technologies.

## Primary Motor Cortex and Basal Ganglia

M1 is located anterior to the central sulcus and can be distinguished from adjacent premotor and somatosensory areas by the presence of a thick cortical descending output layer 5 packed with large pyramidal (Betz) cells and a near‐absent layer 4.^19^ M1 is the target of output from both the basal ganglia and cerebellum and the site where part of the corticospinal descending pathway originates.^20^ The ventrolateral nucleus of the thalamus relays signals from the basal ganglia and provides input to M1, supplementary motor area (SMA), and premotor cortex (PMC).^21^ The SMA, dorsal PMC, and ventral PMC are connected and provide input to M1, while M1 has reciprocal feedback with SMA and dorsal PMC.^21^ Similar to M1, SMA and PMC have projections to the spinal cord through the corticospinal tract.^22^, ^23^ M1 projects back to the basal ganglia primarily to the dorsolateral putamen compared to the input from SMA, which is located more medially in the putamen.^24^ Templates exist to provide brain maps of the cortical motor and premotor regions and the descending tracts for imaging and electrophysiological studies.^22^, ^25^

## Functional Changes in the Motor Cortex of PD

### Positron Emission Tomography (PET)

Nuclear imaging offers the possibility of investigating alterations in cerebral perfusion and metabolism in PD.^17^ A few regional cerebral blood flow (rCBF) studies have shown that during rest the activation pattern in M1 in PD appears to be comparable to that observed in healthy individuals.^26^, ^27^ However, when patients are asked to move, PD patients tend to present with decreased activity in M1,^28^ even when carefully controlling for group differences in kinematics.^29^ Because of the significant cell death in the brain stem nuclei (substantia nigra pars compacta and locus coeruleus), neurochemical signaling in the cortex in PD is thought to be disrupted.^30^ Monoaminergic deficits in M1 were found at rest using Flourine‐18 L‐3,4 Dihidroxyphenylalanine (^18^F‐dopa) PET in a large group of PD and across subgroups with different levels of striatal ^18^F‐dopa uptake.^31^ Newer PET ligands such as (S,S)‐^11^C‐2‐(α‐(2‐methoxyphenoxy)benzyl)morpholine (^11^C‐MeNER) have recently provided the first evidence of a decline in noradrenergic function in M1 in PD that seems to be more pronounced in the advanced stages of the disease.^32^ Together, these studies confirm postmortem immunohistochemical analyses of altered neurochemical projections from the midbrain to M1 in PD^33^ and suggest an involvement of multiple neurotransmitters that could influence future treatments.

The PET literature is not spared, however, from controversy about the direction of functional activity of M1. For instance, a study with PET and Radioactive water ($H_{2}^{15}O$) during a unimanual task in hemiparkinsonian PD found an increased rCBF in M1 compared to controls when patients executed the task with their impaired hand.^34^ Although tasks may influence the pattern of activity, it could well be that specific patterns of activity in M1 are related to different clinical phenotypes. For instance, tremor‐dominant PD patients implanted with a deep brain stimulation (DBS) device in the ventral intermediate thalamic nucleus had increased metabolic activity in M1 and cerebellum in the absence of stimulation, and the level of activity in M1 was positively related to UPDRS ratings of tremor and accelerometer measurements of tremor.^35^ Subsequent thalamic stimulation led to a reduction in the M1 activity and an improvement of tremor ratings. M1 hyperactivity does not only seem to be closely linked to tremor characteristics^36^ but also is a key finding in DBS patients with more severe motor signs, and PD with motor signs affecting other body parts (eg, vocal musculature).^37^, ^38^ Interventions such as intracortical stimulation of STN and noninvasive measures such as vocal training/treatment were successful in modulating the hyperactive state of M1 in these cohorts.^38^, ^39^ Given that restoration of striatal dopamine with oral levodopa provides symptom relief in patients with PD, it becomes imperative to elucidate whether dopaminergic therapy is sufficient for restoring the metabolic state of M1. PET imaging in early stage PD showed that intravenous levodopa infusion could improve UPDRS motor ratings and decrease the regional glucose metabolism in several subcortical and cortical motor structures, including M1,^40^ and that similar results can be obtained with acute oral medication.^41^ However, PD treated chronically with dopaminergic medication have a different response to an acute dose of medication than drug naïve patients, with the latter group having only a small response to the intervention. These results suggest that the severity of the disease as well as chronic exposure to levodopa may influence the responsiveness of M1; however, the mechanisms by which this could happen are not clear.

### Single Photon Emission Computed Tomography

This technique allows the investigation of in vivo metabolic and neurochemical changes^42^, ^43^ and revealed an increase in rCBF in the ipsilateral sensorimotor cortex during a unimanual task in PD with motor fluctuations tested on medication, in the absence of group differences in task performance.^44^ This unexpected finding was attributed to potentially involuntary movements of the other hand that may or may not be induced by levodopa. Interestingly, in a study of PD with levodopa‐induced dyskinesia, M1 was hyperactive bilaterally in patients when compared with controls,^45^ complementing PET findings and suggesting that an overdrive of M1 may underlie hyperkinetic movements (tremor, dyskinesia) in PD. Exploring ways to modulate M1 activity may open therapeutic avenues in PD with drug‐induced dyskinesia.

### Functional Magnetic Resonance Imaging (fMRI)

#### *Task‐Based fMRI*

Functional brain abnormalities in multiple regions of the cortex, including M1, have been reported in several cross‐sectional studies and across a wide range of motor tasks. Reduced functional activity of M1 was found in both drug naïve PD and PD tested off dopaminergic medication using motor tasks such as unimanual grip force production, finger tapping, and finger opposition.^46^, ^47^, ^48^, ^49^, ^50^, ^51^, ^52^, ^53^ These results seem to support the basal ganglia‐thalamo‐cortical circuit model in which dopamine depletion leads to reduced excitatory thalamic outflow to the cortical motor areas which may result in the decrease of functional activity of M1.^54^ Nevertheless, there are a number of studies in drug naïve PD but also PD receiving dopaminergic treatment that have shown an increased activation in M1.^55^, ^56^, ^57^, ^58^ The hyperactivity of M1 tends to be interpreted as a result of reorganization of the motor system to compensate for dysfunction of the basal ganglia. This is an interesting hypothesis worth exploring. However, the longitudinal nature of a process such as compensation makes inferences from cross‐sectional data less robust. An alternative hypothesis is that M1 hyperactivity as detected by fMRI (also PET/SPECT imaging), may be driven by the prevalence of certain motor signs, such as tremor, rigidity, or dyskinesia. It has been long recognized that PD is a heterogeneous disease including hypokinetic and hyperkinectic features.^59^ Among the main subtypes in PD, we have the tremor dominant (TD) and the postural instability and gait difficulty subtypes,^60^ and an fMRI study paired with Electromyography (EMG) measurements showed that PD with tremor presented with increased tremor‐related activity in M1.^61^ Other motor signs such as upper limb rigidity and freezing of gait were also associated with an overactive M1 and suggest that M1 dysfunction is key in the pathophysiology of PD.^55^, ^62^ Similar to PET, task‐based fMRI shows that M1 is responsive to dopaminergic medication,^47^, ^58^ but this effect highlights the importance of a careful control of medication state across patients in functional imaging studies. A less tight control of variables such as the duration of the withdrawal period prior to imaging could influence results in one direction or another (ie, hyperactivity or hypoactivity).

In addition to changes in the magnitude of brain activity in M1, it has become clearer that PD patients also exhibit disruptions of the pattern of functional connectivity between cortical and subcortical motor regions.^63^, ^64^ The abnormal cortico‐subcortical connectivity can be boosted, with recent results demonstrating that an improvement in pedaling rate in PD who had undergone exercise therapy was related to an increase in functional connectivity between M1 and thalamus.^65^ A linear increase in motor‐related connectivity between the putamen and M1 was also detected following levodopa intake in a cohort of PD who later developed levodopa‐induced dyskinesia.^66^ This particular case suggests that an increase in functional connectivity is necessary, but if maintained for a long time could lead to dyskinesia. The plasticity of functional connectivity in response temporary motor practice and acute levodopa highlights the need for more long‐term interventions targeting the cortex that may help relieve some of the motor signs. Collectively, fMRI and task‐fMRI connectivity findings strengthen those obtained with PET by showing that although these measures reflect different characteristics of neural activity, they are feasible for detecting functional abnormalities in M1 that are responsive to intervention.

#### *Resting‐State fMRI*

During the past 2 decades, resting‐state fMRI (rs‐fMRI) has become a popular approach to study abnormalities of spontaneous neuronal activity in the brain in vivo in the absence of task performance.^67^ This method has revealed changes in the cortico‐subcortical functional connectivity, with reduced connectivity between the putamen and M1 in both drug‐naïve and treated PD.^68^, ^69^ Interestingly, the pattern of resting‐state connectivity with the sensorimotor cortex is not the same across the basal ganglia. Unlike the putamen, the STN was consistently found to have increased functional connectivity with M1 in both de novo PD and moderate‐stage PD tested off medication, and the amount of connectivity appears to scale with disease severity.^70^, ^71^ This abnormally increased connectivity between STN and M1 also persists despite the effects of drug therapies, with PD tested on medication presenting a similar interconnectivity pattern, although not as severe.^72^ The temporal correlation of the fMRI signal from the STN and M1 may indicate an overactivity of the hyperdirect pathway, which can be improved by STN stimulation.^73^

Analytic approaches of resting‐state data that examine the functional relationship between remote brain regions (eg, graph theory, seed‐based connectivity) have revealed changes such as decreased nodal centrality that is inversely correlated with UPDRS motor scores, decreased inter‐hemispheric M1 connectivity, and increased functional connectivity with other cortical regions.^74^, ^75^, ^76^ On the other hand, imaging studies that assess changes in the local resting‐state fMRI fluctuations reported reduced regional homogeneity across the basal ganglia as well as M1 and further declines in this measure with disease progression.^77^, ^78^ However, there is also evidence that in some patients there is an increase in regional homogeneity in M1, and this aberrant signal can be normalized with the administration of levodopa.^79^ Furthermore, antiparkinsonian medication was also found to modulate the amplitude of local low‐frequency fluctuations (eg, fALFF) by reducing the initially elevated resting‐state activity detected in the off state.^80^ Together, rs‐fMRI data suggest that M1 in PD is abnormally engaged in both long‐distance connections with remote regions and short‐distance connections.

### Electroencephalography (EEG)

Brain activity is characterized by synchronized oscillations between populations of neurons, and cortical motor regions such as M1 are known for having prominent oscillations in the alpha and beta frequency band (ie, 8‐12 Hz, 13‐30 Hz).^81^, ^82^ Typically when a motor task is performed there is an attenuation of brain oscillations in the alpha and beta bands, and this process is known as desynchronization.^83^ In PD, a number of studies revealed an aberrant pattern of synchronization/desynchronization in the beta rhythm across the basal ganglia‐thalamo‐cortical circuit.^81^, ^84^, ^85^, ^86^ It is believed that the parkinsonian brain is characterized by an impairment in switching between akinetic (beta band) and prokinetic (gamma band) oscillations in the sensorimotor cortex of PD patients,^82^ and abnormal levels of beta activity within and between the cortex and basal ganglia that correlate with motor deficits but respond to interventions.^87^, ^88^, ^89^ For instance, high‐frequency stimulation of STN in PD reduced STN beta activity and decreased the sensorimotor cortical–STN coherence in the beta band.^89^ Acute treatment with antiparkinsonian medication is also successful as it was found to attenuate the alpha and beta rhythms over the cortical motor areas during wrist movements, and this effect correlated with improvements in bradykinesia.^90^ Positive effects of medication were also observed in the level of desynchronization in M1 prior to movement.^91^ Together, these studies suggest that abnormal beta oscillations may indeed underlie not all but many of the motor deficits observed in PD. One of the motor states least understood in PD is levodopa‐induced dyskinesia. A study focusing on a rodent lesion model of PD found that dyskinesia was always accompanied by a strong narrowband oscillation at ∼80 Hz in the motor cortex of the lesioned hemisphere.^92^ Interestingly, data collected for 1 year in 2 PD patients permanently implanted with an electrocorticography strip recording potentials over M1 found similar results with dyskinesia being associated with a narrowband gamma oscillation in M1 between 60 and 90 Hz.^93^ Collectively, studies of cortical oscillations in PD point to perturbed low‐frequency oscillatory activity within motor regions that could serve as a potential therapeutic target in future intervention studies.

### Magnetoencephalography (MEG)

MEG can record task‐related activity in the brain as well as brain activity at rest. Using MEG, it has been shown that PD patients have increased sensorimotor cortical power in the beta band frequency compared to controls at rest and during upper limb movements.^94^ Moreover, MEG recordings confirm EEG findings showing abnormal beta desynchronization occurring not only during movement but also prior to and after movement.^95^ Based on coherence estimates derived from simultaneous multisite cortical MEG and local field potential recordings from STN, we know that there is a strong coherence in beta oscillations between the sensorimotor cortex and the basal ganglia, and between the sensorimotor cortex and other cortical motor regions.^96^ Acute dopaminergic medication appears to reduce this abnormal coupling.^97^ Brain activity in M1 was found to be coherent not only with activity of other regions but also with tremulous EMG, suggesting that abnormal oscillations in M1 contribute to resting tremor in PD.^98^ MEG results complement findings derived from other imaging modalities (PET, fMRI) showing that M1 hyperactivity is important in a tremor‐related network.

## Structural Changes in the Motor Cortex of PD

### Morphometry

**Figure 1:**
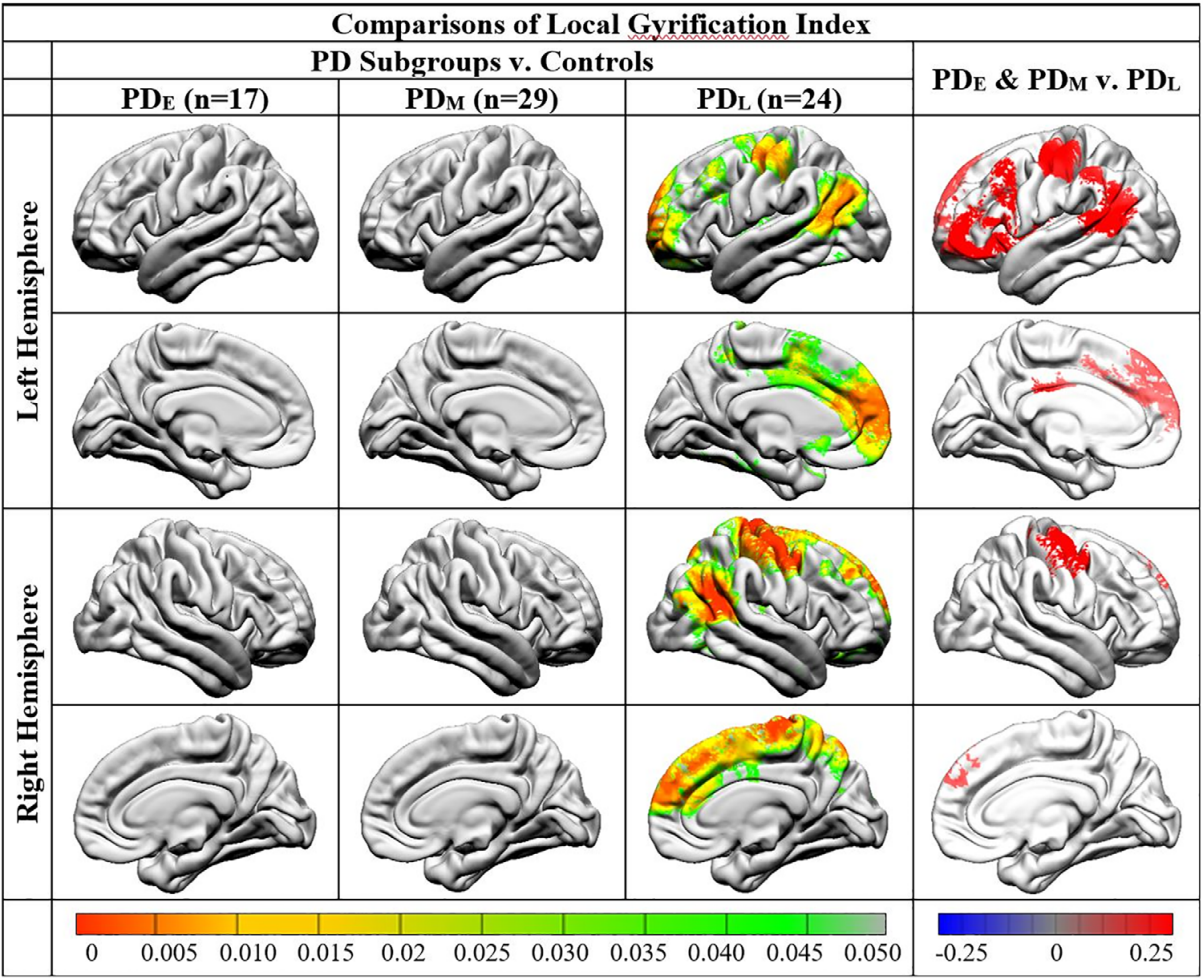
Comparison of local gyrification index between Parkinson disease (PD) subgroups and controls (left) and among PD subgroups (right) at baseline. In this study, PD patients were grouped based on the number of years since diagnosis into PD‐early (PE_E_; < 1 year, baseline UPDRS‐III on medication of 11.4 ± 8.2, baseline Hoehn and Yahr stage 1.4 ± 0.6), PD‐middle (PE_M_; 1‐5 years, baseline UPDRS‐III on medication of 19.8 ± 10.4, baseline Hoehn and Yahr stage 1.4 ± 0.6), and PD‐late (PE_L_; > 5 years, baseline UPDRS‐III on medication of 20.4 ± 11.9, baseline Hoehn and Yahr stage 2.2 ± 0.6). PD with a long disease duration had reduced gyrification bilaterally in several cortical areas including M1 compared to controls at baseline (left). These patients also had reduced gyrification in several neocortical areas when compared with PD with short and medium disease duration at baseline (right). Figure reprinted with permission from Sterling NW, Wang M, Zhang L, et al. Stage‐dependent loss of cortical gyrification as Parkinson disease “unfolds”. Neurology 2016;86(12):1143‐1151.^109^

Three‐dimensional–T1 weighted MRI is widely used to study changes in brain structure in various neurological diseases as it provides good gray–white matter contrast. In diseases that affect the basal ganglia, this type of sequence is more challenging because the contrast of many nuclei is relatively poor because of the rich iron content, making accurate delineation of these structures difficult.^9^, ^99^ From a methodological standpoint, assessing cortical changes in PD using T1‐based morphometry methods is less challenging. Using voxel‐based morphometry of gray matter we have learned that cortical morphometry in nondemented PD patients is typically normal or with minor differences when compared with controls.^9^, ^99^, ^100^ By contrast, in PD with mild cognitive impairment or dementia, there are more robust gray matter changes in the parieto‐temporal regions. ^9^ Changes in cortical motor regions are not very common, with a few observations of reduced gray matter volume in the precentral gyrus in PD with cognitive impairment^101^, ^102^, ^103^, ^104^ and in the postural instability and gait difficulty PD subtype.^105^ Alternatives to voxel‐based morphometry that allow the estimation of gray matter changes (eg, surface‐based morphometry),^106^, ^107^ have revealed cortical thinning in the sensorimotor cortex in nondemented PD when compared with controls and reduced cortical gyrification in M1 in those PD patients with more advanced disease duration (Fig. 1).^108^, ^109^ Although based on the majority of T1‐based studies structural changes in M1 are not a signature of PD, the latest results using surface‐based morphometry raise the possibility that changes in structure could occur once patients reach the more moderate to advanced stages of the disease and may explain emerging signs such as the progressive loss of balance and increasing gait difficulty. Gray matter changes as picked up by the current macrostructural methods may be small and evolve slowly, highlighting the need for longitudinal designs and the development of more sensitive metrics.

### Diffusion MRI

Diffusion MRI can be used to quantify microstructural changes in parts of the brain that are unremarkable on routine structural MRI scans.^110^ Fractional anisotropy (FA) is a common index derived from diffusion MRI data that reflects the degree of anisotropic motion of water molecules within a voxel that can be modulated by the complex brain tissue environment.^111^ In PD, it has been widely used in evaluating structural changes in the substantia nigra,^112^, ^113^, ^114^, ^115^, ^116^ but its applications are not restricted to subcortical structures. Reduced FA was detected in the precentral gyrus and SMA in PD, whereas another study assessing diffusion MRI changes across motor tracts found increased FA in the corticospinal and thalamus‐motor cortex tracts in patients when compared with controls.^117^, ^118^ These rather unexpected findings highlight the possibility of microstructural changes being far more extensive than previously thought, encompassing complex pathways involved in the initiation and control of movement. However, future cross‐sectional and longitudinal diffusion MRI studies are needed to confirm these observations and assess their relation to motor signs in PD. At the same time, we recommend caution in interpreting the significance of cortical motor changes because diffusion metrics such as FA, although sensitive to microstructural changes, lack specificity with respect to the underlying biological mechanisms (eg, neuronal loss, compensatory axonal sprouting, changes in myelination and fiber density, neuroinflammation).^119^ For now, determining whether the microstructural changes detected in M1 and the motor tracts based on diffusion MRI are neurodegenerative or compensatory in nature remains an important challenge.

## PD Progression and Changes in the Motor Cortex

**Figure 2:**
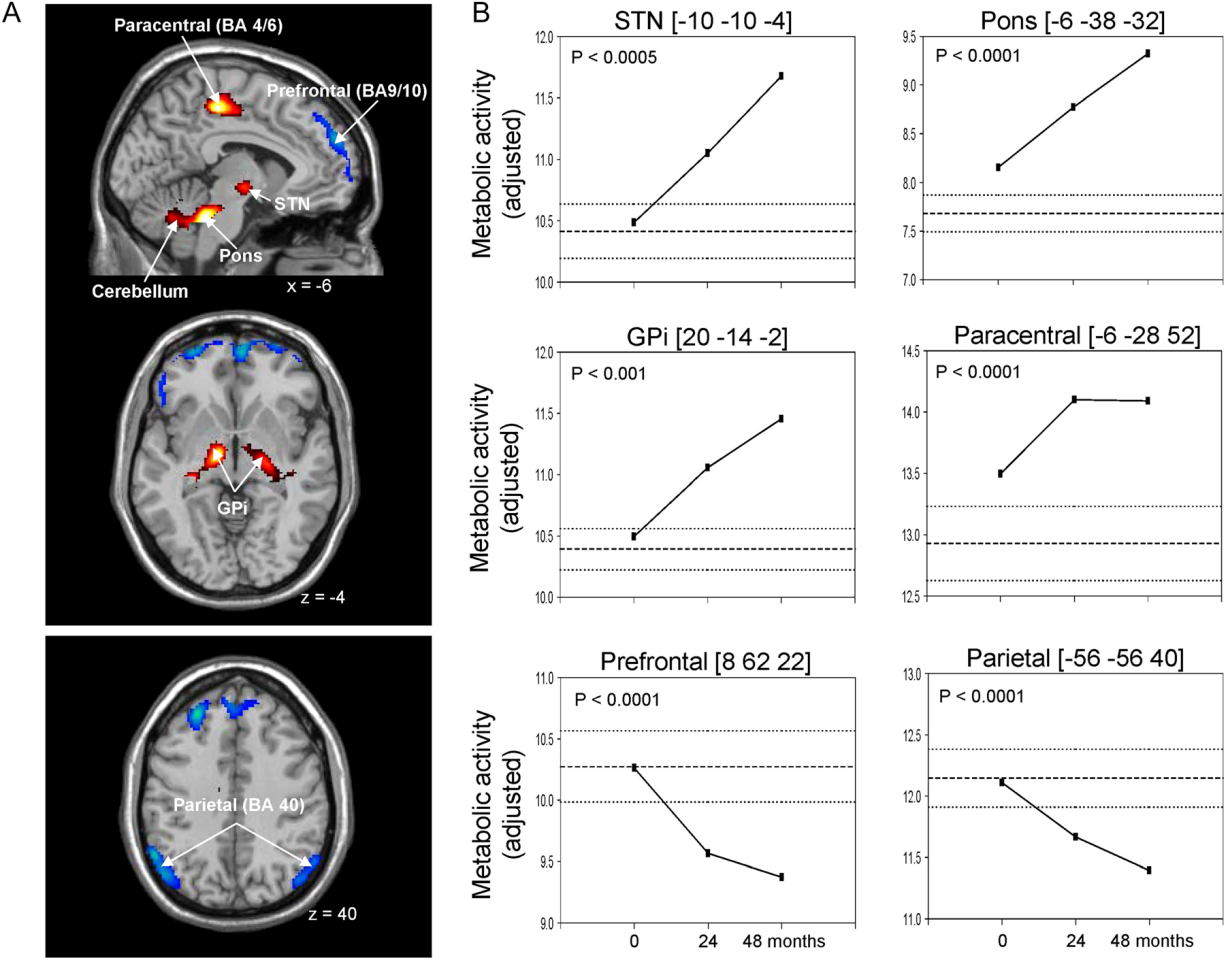
(**A**) Voxel‐based analysis of longitudinal changes in regional metabolic activity. Metabolic increases with disease progression are displayed using a warm colors. Progressive metabolic declines are displayed using a cold colors. (**B**) metabolic data at each timepoint. The coordinates refer to the Montreal Neurological Institute (MNI) standard space. GPi, internal globus pallidus; STN, subthalamic nucleus; BA, Brodmann area. Figure reprinted with permission from Huang C, Tang C, Feigin A, et al. Changes in network activity with the progression of Parkinson's disease. Brain 2007;130(7):1834‐1846.^120^

**Figure 3:**
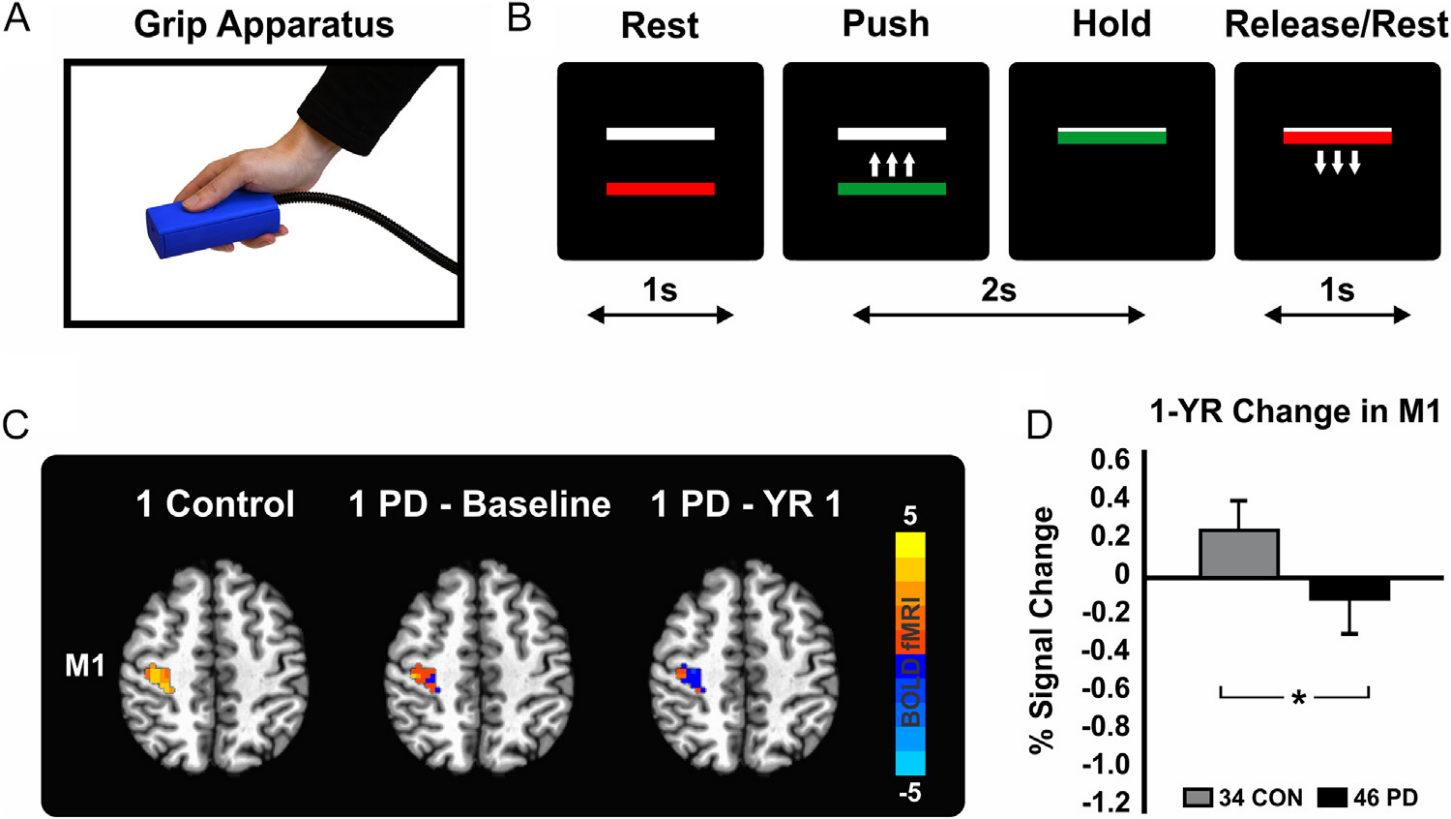
(**A**) Grip apparatus used to produce force. PD patients performed the task with the more affected hand. (**B**) Task consisting of a series of 2 seconds of force production and 1 second of rest. Force target was set at 15% of maximum voluntary contraction. (**C**) Functional MRI signal during grip force production in the contralateral M1 in 1 healthy control along with task‐based fMRI signal at baseline and 1 year later in one PD patient. (**C**) Group statistics showing a reduction in force‐related fMRI activity in M1 in PD during the course of 1 year. Data represent the 1‐year difference adjusted for the following variables at baseline: age, sex, Montreal Cognitive Assessment Test, and percent signal change in M1. CON, controls; M1, primary motor cortex; PD, Parkinson's disease; YR, year. Figure adapted from Burciu RG, Chung JW, Shukla P, et al. Functional MRI of disease progression in Parkinson disease and atypical parkinsonian syndromes. Neurology 2016;87(7):709‐717.^122^

Although the PD neuroimaging literature addressing longitudinal changes in M1 is limited, it is crucial to gain a better understanding of the rate of progression of M1 changes to develop therapeutic approaches. A 4‐year multitracer PET imaging study in early‐stage PD patients revealed time‐dependent increases in metabolism in several brain regions including M1 (Fig. 2).^120^ Unlike other regions with a steady increase in glucose consumption, the regional metabolism in M1 did not rise continuously over time, but plateaued after 2 years. An interesting observation was that tremor ratings followed a similar pattern that suggests that the observed metabolic changes in M1 may reflect progression of specific motor signs such as tremor. Longitudinal changes in M1 were also detected in fMRI studies. Cohorts of PD patients followed up for 2 years presented with a decline at rest in the local amplitude of low‐frequency fluctuations and regional synchrony of low‐frequency fluctuations.^78^, ^121^ The level of activity of M1 during movement also deteriorated over time, with PD followed up for 1 year showing reduced fMRI signal compared to controls (Fig. 3).^122^ The results were observed in a large sample and in the absence of any group differences in task performance. The lack of a time effect in the control group highlights the potential of task‐based fMRI as a progression marker that could aid in testing therapeutic strategies aimed at slowing down the progression of the disease. Furthermore, if confirmed, the sensitivity of task‐based fMRI to the progression of PD may hold promise for the prodromal phase of the disease. It is known that brain changes in PD begin long before the onset of clinical signs, and during the prodromal period individuals present with a combination of subtle motor and nonmotor signs that are currently not sufficient to diagnose the disease.^123^ Based on the involvement of M1 in the pathophysiological profile of PD, it could be that people at risk for PD present with abnormalities in this structure in the absence of overt motor signs. A recent multimodal imaging study of healthy adults who do not have motor symptoms but carry a risk genotype/variant in the α‐synuclein gene (SNCA rs356219) associated with increased odds of developing PD, revealed functional changes in the brain of carriers that mimic those observed in patients with PD (ie, M1 hypoactivity).^124^ Changes in M1 were also found in prodromal individuals with Rapid eye movement (REM) sleep behavior disorder followed up clinically to assess PD conversion.^125^ The PD‐related covariance pattern of metabolic activity at rest known to include different patterns of activity such as increases in M1 was elevated in the group at risk for PD.

As for structural imaging measures, these may also help gauge disease progression in PD. A recent 3‐year progression study of PD without dementia showed reduced cortical gyrification in the precentral and postcentral areas in more advanced stages of the disease and a decline in this measure with disease progression.^109^ Although structural changes in M1 are not a hallmark of PD, they raise the hypothesis that functional cortical changes in PD could be followed by structural changes. In a study that compared cortical metabolism and measures of cortical atrophy in PD with intact cognition, PD with mild cognitive impairment, and PD with dementia, the results showed a gradient in cortical changes that suggests that functional changes precede structural changes.^104^ Together, the existing longitudinal studies reinforce the importance of M1 in the progression of the disease. The development of new treatment options (whether pharmacological or not) is important because dopaminergic medication is not sufficient to slow down changes in M1.

## Imaging the Parkinsonian Brain: What Have We Learned About the Motor Cortex?

Collectively, human brain imaging studies have proven useful in investigating the many facets of PD, contributing to a better understanding of the pathophysiological changes in M1. A summary of the main findings obtained with different imaging techniques can be found in Supplementary Figure 1. Importantly, M1 abnormalities in PD are often functional in nature, emerge across a wide range of motor paradigms and at rest, across all stages of the disease, and are partially restored by interventions. Structural changes in M1 are not as common and seem to be present in a subgroup of patients who are either in a more advanced stage of the disease or present with additional signs (eg, posture and gait instability, cognitive deficits). When the functional neuroimaging studies are examined as a whole, 2 activation patterns in M1 emerge (ie, hypoactivation vs hyperactivation). When interpreting these patterns, one should keep in mind the following important issues in the functional imaging field: group differences in task performance and how much the task controls behavioral output. In experiments that do not account for task performance, any difference in activation observed between controls and PD might be the result of a less successful performance of the patient group rather than a fundamental difference in the way the parkinsonian brain processes the motor task. For instance, parameters such as rate and amplitude of movements and the number of iterations within the measured interval can vary between PD and controls, which may account for differences in activation patterns. In button‐pressing tasks, the force applied to the button is not measured, and thus PD could be pressing with greater force and this would not be known. It is well known that increasing muscle force increases firing rates of pyramidal tract neurons in M1.^126^ Also, functional imaging experiments have shown that increasing force is associated with increases in fMRI signal and rCBF in M1.^127^, ^128^ In a joystick‐movement task, commonly used in fMRI, the experimenter does not know if the amount of force applied to the joystick is similar between patients and controls. However, records of joystick movements can be analyzed and integrated in group statistics. For instance, in the PET study of Turner and colleagues^29^ examining brain activity in PD during a visuomotor tracking task, movement velocity was used as a covariate to factor out major between‐group differences in performance. Their analysis revealed several regions involved in motor control, including M1, had reduced activity in PD.^29^ Similarly, in studies that do use a force control task and the level of force is controlled along with the duration and number of force pulses, there is a highly consistent and replicated pattern of reduced fMRI activity in the M1 for PD patients when compared with controls.^48^, ^49^, ^50^, ^51^, ^53^, ^122^ In general, across PET and task‐based fMRI modalities, the common observation is that the activity of M1 during movement in those studies that controlled the way the task was executed was reduced in PD patients when compared with controls. However, there are also several studies that reported increases in metabolic activity as well as fMRI signal in PD patients when compared with controls. Of particular importance is that hyperactivity of M1 both at rest and during movement seems to be specific to a certain clinical phenotype, PD with tremor or dyskinesia. An important aspect that one has to bear in mind when interpreting patterns of activity in M1 is the fact that M1 is the target of somatotopically organized outputs from both the basal ganglia and cerebellum.^20^ Although it is not clearly understood how the basal ganglia and cerebellum interact with each other, there is increasing evidence on the involvement of the cerebellum in resting tremor in PD,^61^, ^129^ and it could be that information processing along this parallel pathway connecting the cerebellum to cortical motor areas contributes to and modulates the pathophysiological state of M1 in PD. Finally, although the existing dichotomy of functional activity in M1 as revealed by nuclear imaging and MRI may seem somewhat contradictory, it also raises the question whether PD may be associated with a patterned combination of hypo‐ and hyperactivity. However, the current imaging data acquired with different methods and spatial resolutions cannot provide an answer. Another open and interesting question that remains to be answered is whether the overdrive of M1 as detected by functional imaging in some cohorts of PD could be a form of early compensation that counteracts the effect of low dopamine levels in the brain, but longitudinal studies in early PD are needed to address this issue.

From the other functional imaging modalities (rs‐fMRI, EEG, and MEG), we have learned more about the different properties of the motor brain network as a whole in PD. Although they differ in temporal and spatial resolution, by corroborating information derived from these different techniques, it becomes clearer that changes in PD in M1 are complex and can be characterized by abnormalities in the beta‐band synchronization, local and long‐range coherence of neural oscillations, and dynamics of spontaneous Blood oxygen level dependent (BOLD) fluctuations. Although data support the pathological neural oscillations model,^130^, ^131^ they also suggest that changes in motor signs could result from a conglomerate of abnormalities ranging from changes in firing rate, pattern, and synchrony of neural populations.

As for structural changes in M1, it is worth noting that current models of PD assume the absence of any overt pathology in cortical motor regions. Although there is not sufficient evidence on structural abnormalities in M1 in PD based on noninvasive imaging, a few findings raise an intriguing hypothesis that M1 dysfunction in PD is not exclusively a consequence of basal ganglia malfunctioning. Prior work has reported microstructural changes in PD including Lewy bodies in the Betz cells of M1 and a reduction in dopaminergic innervation to layer 1.^33^, ^132^ Whether or not structural changes emerge later in the disease, it is plausible to assume that structural changes could have an impact on M1 activity independently of any modulatory changes coming from the basal ganglia. At the same time, the presence of structural changes in M1 as detected by structural MRI highlights an important issue in the field and that is the reliability of diagnosis in PD. The clinical diagnosis in PD is still challenging as a result of difficulties in recognizing atypical parkinsonian syndromes early on.^133^ Atypical parkinsonian patients present with extensive cortical atrophy including changes in motor regions when compared to PD,^134^, ^135^ so the accuracy of diagnosis should be considered when interpreting structural data. Every effort must be made to ensure that research studies recruit PD who have had a stable diagnosis. Also, the body of existing literature on cortical changes in PD suggests that many of these changes are related to a variety of clinical signs that prompt the need to address heterogeneity of PD in imaging studies, and validation on these findings in larger cohorts of subjects. In general, a better understanding of what sometimes appears to be conflicting evidence may be resolved by a careful evaluation of the research cohorts, the presence or absence of a control group, sample size, heterogeneity of motor signs, duration of withdrawal from antiparkinsonian medication that could alter the degree of functional activity of M1, statistical methods, and limitations of the imaging modalities.

In summary, a variety of neuroimaging techniques provide complementary information and demonstrate predominantly functional changes in M1 in PD and prompt for more research geared toward the development of therapeutics and interventions that go beyond replenishing dopamine depletion and target the motor cortex, potentially slowing disease progression.

## Author Roles

1) Research project: A. Conception, B. Organization, C. Execution; 2) Statistical Analysis: A. Design, B. Execution, C. Review and Critique; 3) Manuscript: A. Writing of the first draft, B. Review and Critique.

R.G.B.: 3A, 3B

D.E.V.: 3A, 3B

## Full financial disclosure for the previous 12 months

D.E.V. reports grants from the National Institutes of Health, National Science foundation (NSF), and Tyler's Hope Foundation during the conduct of the study and personal honoraria from National Institutes of Health, National Parkinson's Foundation, and Northwestern University unrelated to the submitted work. He is Co‐Founder of Neuroimaging Solutions, LLC. R.G.B. reports no disclosures.

## Supporting information


**Figure S1**
