# Supplementary figures and images for: Imaging of Motor Cortex Physiology in Parkinson's Disease
Source: Mov Disord. 2018 Oct 2;33(11):1688–99. doi: 10.1002/mds.102 (PMC6261674; doi:10.1002/mds.102)

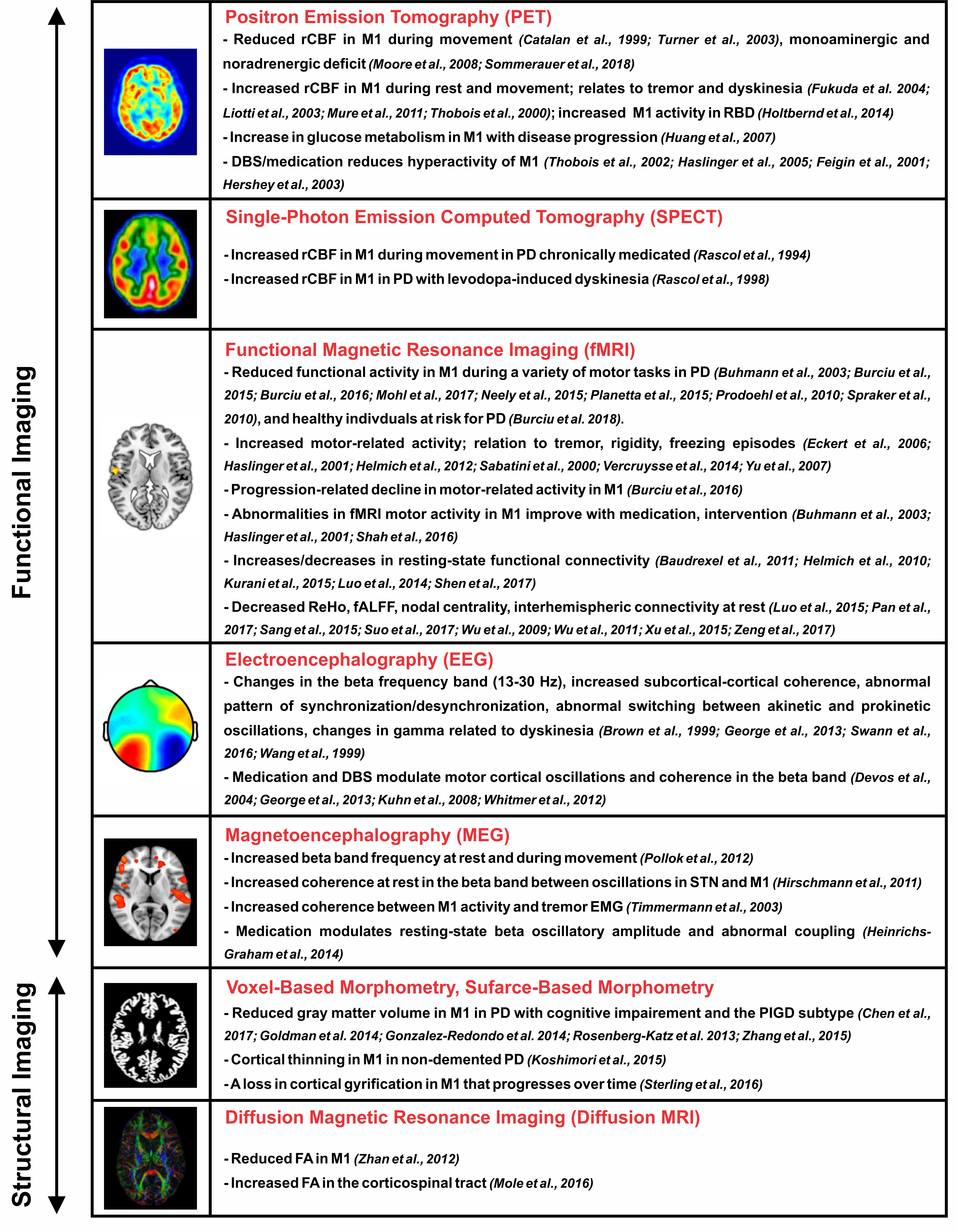

Supplement: Supplementary file 1 — Figure S1 [file MDS-33-1688-s001.tiff]
